# The screening and prediction of functional/bioactive compounds in foods: Editorial; how to efficiently discover functional/bioactive compounds in foods?

**DOI:** 10.1002/fsn3.3146

**Published:** 2022-11-15

**Authors:** Jingli Xie

**Affiliations:** ^1^ State Key Laboratory of Bioreactor Engineering, Department of Food Science and Technology East China University of Science and Technology Shanghai China

Nowadays, with the research progresses of life science, such as the understanding of the effects of gut microbiota on human health, the discovery of new drug targets, and the application of synthetic biology in natural compounds production, the roles of foods are not only limited to alleviating hunger and fulfilling nutrition, but also in favor of maintaining health, ameliorating subhealth and preventing disease.

In addition to common nutrients, plant foods also contain a large number of bioactive compounds polyphenol, flavonoids, alkaloid, terpenoids, steroids, quinonoids, glycosides, and pigments. These bioactive compounds have become research focus for decades, and been proved to have functional activities such as antioxidant, antiinflammation, antihypertension, antimicrobial, and antitumor after they were taken. For animal foods, such kinds of bioactive substances were seldom found. However, abundant proteins with various sequences may provide massive peptide fragments through digestion or artificial hydrolysis. A lot of food‐borne peptides have been reported to display amounts of bioactivities, for instance, antihypertension, blood sugar lowering, antimicrobial, antioxidant, immunomodulation, and opioid agonist.

Due to the complicated composition of food‐stuff, the discovery of food borne bioactive compounds via laboratory work is time‐ and labor‐consuming. Usually, the extraction, separation and purification are the routine protocols to obtain a certain compound from foods. For the bioactive peptides, digestion or hydrolysis is essential step. The loss of potential candidates during the extraction, separation, and purification, as well as the incomplete digestion are the bottlenecks for the discovery efficiency. Therefore, high‐throughput techniques are pressing to be developed.

Thanks to the application of Informatic and Computing Technology in Biology and Pharmacy, people have thereby proposed lots of tools to help themselves liberate from heavy lab work and transform the field to the computer. Lots of online databases, such as BLAST, PDB, CAZyDB, BioPEP, as well as docking tools, such as GOLD, Discovery Studio, AutoDock, SwissDock, BSP‐SLIM, 1‐CLICK DOCKING. Recent years, techniques of the protein structure prediction were also booming. ROBETTA, I‐TASSER, sAlphaFold, RaptorX appeared. Especially, AlphaFold took the lead in reaching atomic accuracy as 60% in 2018, and then AlohaFold 2 realized the accuracy more than 90% in 2020, which was so exciting for the people working in silico that the artificial protein could be successfully expressed.

The virtual screening was initiated by pharmacy scientists whom strived to find drugs from thousands candidate molecules. Researchers in Food Science have also concerned the collaboration of computing techniques and screening/prediction of functional/bioactive compounds in foods. For example, if we use the traditional process to isolate a novel bioactive peptide, we need hydrolyze the protein first, and then cut the hydrolysate according to the molecular weight, and determine the activity of each portion, and continue to separate the portion with the highest activity, and then test the activity of each fraction…till we get a peptide verified by Mass spectrum, which takes at least several months for a skilled worker. Nevertheless, when the in silico procedure is used, we can harvest more bioactive peptides even in a week. Besides, more and more mechanism illustrations or structure–activity relationships were elucidated in the way of molecular docking, which brought about more visual description of the interactions between the ligand and the receptor.

Accordingly, the present special issue will focus on the new techniques of screening, prediction and design of functional/bioactive compounds in foods, including high‐throughput methods, virtual/in silico strategies, as well as the novel techniques in the assay of the bioactivities in silico, in vitro or in vivo, or the illustration of molecular mechanism.

